# Demographic Factors Associated With Non-Guideline–Based Treatment of Kidney Cancer in the United States

**DOI:** 10.1001/jamanetworkopen.2021.12813

**Published:** 2021-06-09

**Authors:** Jeffrey M. Howard, Karabi Nandy, Solomon L. Woldu, Vitaly Margulis

**Affiliations:** 1 Department of Urology, University of Texas Southwestern Medical Center, Dallas; 2 Department of Population and Data Sciences, The University of Texas Southwestern Medical Center, Dallas

## Abstract

**Importance:**

Significant demographic disparities have been found to exist in the delivery of health care. Demographic factors associated with clinical decision-making in kidney cancer have not been thoroughly studied.

**Objective:**

To determine whether demographic factors, including sex and race/ethnicity, are associated with receipt of non-guideline–based treatment for kidney cancer.

**Design, Setting, and Participants:**

This retrospective cohort study was conducted using data from the National Cancer Database for the years 2010 through 2017. Included patients were individuals aged 30 to 70 years with localized (ie, cT1-2, N0, M0) kidney cancer and no major medical comorbidities (ie, Charlson-Deyo Comorbidity Index score of 0 or 1) treated at Commission on Cancer–accredited health care institutions in the United States. Data were analyzed from November 2020 through March 2021.

**Exposures:**

Demographic factors, including sex, race/ethnicity, and insurance status.

**Main Outcomes and Measures:**

Receipt of non-guideline–based treatment (undertreatment or overtreatment) for kidney cancer, as defined by accepted clinical guidelines, was determined.

**Results:**

Among 158 445 patients treated for localized kidney cancer, 99 563 (62.8%) were men, 120 001 individuals (75.7%) were White, and 91 218 individuals (57.6%) had private insurance. The median (interquartile range) age was 58 (50-64) years. Of the study population, 48 544 individuals (30.6%) received non-guideline–based treatment. Female sex was associated with lower adjusted odds of undertreatment (odds ratio [OR], 0.82; 95% CI, 0.77-0.88; *P* < .001) and higher adjusted odds of overtreatment (OR, 1.27; 95% CI, 1.24-1.30; *P* < .001) compared with male sex. Compared with White patients, Black and Hispanic patients had higher adjusted odds of undertreatment (Black patients: OR, 1.42; 95% CI, 1.29-1.55; *P* < .001; Hispanic patients: OR, 1.20; 95% CI, 1.06-1.36; *P* = .004) and overtreatment (Black patients: OR, 1.09; 95% CI, 1.05-1.13; *P* < .001; Hispanic patients: OR, 1.06; 95% CI, 1.01-1.11, *P* = .01). Individuals who were uninsured, compared with those who had insurance, had statistically significantly higher adjusted odds of undertreatment (OR, 2.63; 95% CI, 2.29-3.01; *P* < .001) and lower adjusted odds of overtreatment (OR, 0.72; 95% CI, 0.67-0.77; *P* < .001).

**Conclusions and Relevance:**

This study found that there were significant disparities in treatment decision-making for patients with kidney cancer, with increased rates of non-guideline–based treatment for women and Black and Hispanic patients. These findings suggest that further research into the mechanisms underlying these disparities is warranted and that clinical and policy decision-making should take these disparities into account.

## Key Points

**Question:**

Are demographic factors, such as sex, race/ethnicity, and insurance status, associated with deviation from guideline-based treatment of kidney cancer?

**Findings:**

In this cohort study of 158 445 patients with localized kidney cancer in the National Cancer Database, female patients were treated more aggressively compared with male patients, with lower adjusted odds of undertreatment and higher adjusted odds of overtreatment. Black and Hispanic patients had higher adjusted odds of undertreatment and overtreatment compared with White patients, and uninsured status was associated with lower adjusted odds of overtreatment and higher adjusted odds of undertreatment.

**Meaning:**

These findings suggest that sex, race/ethnicity, and insurance status are associated with significant disparities in the receipt of guideline-based treatment for localized kidney cancer.

## Teaser

This cohort study investigates the association of sex, race/ethnicity, and insurance status with undertreatment and overtreatment of kidney cancer.

## Introduction

The past several decades have seen an increased incidence and downward stage migration of kidney cancer. These phenomena have been driven by incidental detection of small kidney masses on nonurologic cross-sectional imaging, in what one set of authors described as “nonsystematic partial screening of the population.”^1^ It has been recognized that small (ie, <4 cm) tumors in the kidney often have low metastatic potential and that patients with these tumors are often more likely to die of competing causes.^2,3^ As a result, major organizations have issued guidelines favoring nephron-sparing approaches (ie, partial nephrectomy or ablation) or active surveillance in patients with small kidney masses.^4,5,6^

Although the overtreatment of clinically indolent lesions is a recognized and important phenomenon in urologic oncology, increasing attention has also been drawn to undertreatment of clinically aggressive cancers, particularly among underserved racial/ethnic groups and among patients who are uninsured or underinsured.^7,8,9^ However, the association between patient demographic characteristics and clinical decision-making in kidney cancer remains poorly studied. In this study, we sought to assess the association between demographic factors (including sex, race/ethnicity, and insurance status) and receipt of non-guideline–based kidney cancer treatment in a comparatively young and healthy cohort of patients with localized kidney cancer and low competing risk of mortality.

## Methods

This was a retrospective cohort study using data from the National Cancer Database (NCDB). The NCDB is a joint project of the Commission on Cancer (CoC) of the American College of Surgeons and the American Cancer Society. It is a hospital-based registry containing data from more than 1500 CoC-accredited hospitals in the United States and includes approximately 70% of all new cancer diagnoses in the United States.^10^ This study was exempted from review by the University of Texas Southwestern Medical Center (UTSW) institutional review board (IRB) owing to the use of deidentified patient data. Informed consent was waived by the UTSW IRB owing to the minimal risk to patients and the inability to identify specific patients in the deidentified data set. The Strengthening the Reporting of Observational Studies in Epidemiology (STROBE) reporting guideline was followed.

### Study Population

The NCDB contained 339 641 patients with a primary diagnosis of kidney cancer (using *International Statistical Classification of Diseases and Related Health Problems, Tenth Revision *[*ICD-10*] site code C64.9) from January 1, 2010, to December 31, 2017. These dates were chosen to coincide with the implementation of the 2009 American Urological Association (AUA) guideline on management of small kidney masses.^11^ Of these patients, 214 763 patients were aged 30 to 70 years and had a Charlson-Deyo Comorbidity Index (CCI) score of 0 or 1. We excluded patients who had documented metastatic disease or were missing information on metastatic status; we then performed multiple imputation of missing values as described in the eAppendix in the Supplement. (Missing values are listed in eTable 1 in the Supplement.) After additional exclusions, the final study population consisted of 158 445 patients with cT1-2, N0, M0 kidney cancer (eFigure in the Supplement). Cancer staging was according to the seventh edition of the American Joint Committee on Cancer TNM system.^12^

### Definition of Guideline-Based Treatment

Our definition of guideline-based treatment was based on the National Comprehensive Cancer Network (NCCN) clinical practice guideline version 1.2021, which incorporates tumor size and clinical stage.^6^ In this guideline, surveillance or nephron-sparing approaches (ie, ablation or partial nephrectomy) are favored in smaller noninvasive masses, whereas radical nephrectomy is favored in larger masses. Patients were assigned to 1 of 4 tumor classifications based on tumor size and clinical stage data. Treatments were categorized as observation or surveillance (including any patient with ≥6 months elapsed from diagnosis to treatment), ablation (eg, cryosurgery or thermal ablation), partial nephrectomy, and radical nephrectomy. Patients were then categorized as receiving guideline-based treatment, undertreatment, or overtreatment according to the scheme shown in eTable 2 in the Supplement. These designations were intended to allow broad categorization of treatment choices among patients without implying judgment of appropriateness for any individual patient.

### Statistical Analysis

The primary outcome of interest was receipt of guideline-based or non-guideline–based treatment (ie, undertreatment or overtreatment). Rates of non-guideline–based treatment were assessed across demographic and institutional variables using the Pearson χ^2^ test for categorical variables and Kruskal-Wallis 1-way analysis of variance on ranks for continuous variables. A multinomial logistic regression model was fitted for the outcome with all demographic and institutional factors as independent variables. Finally, subgroup analysis was performed by stratifying the study population by tumor classification (as used to define guideline-based treatment) and performing binary logistic regression. This allowed us to account for differential distribution of tumor size across populations. Regression models included the following covariates: age, year of diagnosis, sex, race/ethnicity, CCI score, insurance status, facility type, facility location, facility annual patient volume (by quartile), urban vs rural location, distance traveled to facility (by quartile), and median income and educational attainment of patient’s home zip (postal) code. Patient sex and race/ethnicity were abstracted from medical records by the participating institutions and were therefore dependent on documentation practices of individual treating institutions, which could not be determined with certainty. Race/ethnicity was assessed because this information is required by the NCDB. Multicollinearity was tested by calculation of variance inflation factors (eTable 3 in the Supplement). Sensitivity analyses (eAppendix in the Supplement) included repeating the analysis on a nonimputed data set with missing values excluded and conducting subgroup analyses with tumor size included as a continuous covariate (eTable 4 and eTable 5 in the Supplement). The threshold for significance was set at *P* ≤ .05. All reported *P* values were 2-tailed. All analyses were performed using SPSS statistical software version 27 (IBM Corp) from November 2020 through March 2021.

## Results

### Patient Characteristics

Patient characteristics and receipt of guideline-based treatment are presented in Table 1. By intent, our population represented a comparatively young and healthy cohort among whom advanced age and comorbidity were unlikely to be associated with treatment decisions. The median (interquartile range) age was 58 (50-64) years, 36 723 individuals had a CCI score of 0 (23.2%), and 121 721 individuals (76.8%) had a CCI score of 1. There were 99 563 (62.8%) men, 120 001 White individuals (75.7%), and 91 218 individuals with private insurance (57.6%). Overall, 109 901 patients (69.4%) received guideline-based treatment and 48 544 patients (30.6%) received non-guideline–based treatment, among whom 3893 patients (2.5%) were undertreated and 44 651 patients (28.2%) were overtreated. The total number of patients increased over time, and the proportion of patients receiving guideline-based treatment increased over the study period, from 11 206 of 16 934 (66.2%) in 2010 to 15 055 of 21 126 patients (71.3%) in 2017 (*P* < .001) (Table 1).

**Table 1.** Patient Characteristics

Characteristic	Patients, No. (%)^a^				*P* value^b^
	Receiving undertreatment	Receiving guideline-based treatment	Receiving overtreatment	Total	
Year^c^					
Total	3893 (2.5)	109 901 (69.4)	44 651 (28.2)	158 445 (100)	
2010	355 (2.1)	11 206 (66.2)	5373 (31.7)	16934 (10.7)	<.001
2011	371 (2.1)	12 099 (67.8)	5382 (30.1)	17852 (11.3)	
2012	441 (2.3)	12 927 (68.3)	5559 (29.4)	18927 (11.9)	
2013	503 (2.6)	13 660 (69.5)	5479 (27.9)	19642 (12.4)	
2014	507 (2.4)	14 512 (69.8)	5759 (27.7)	20778 (13.1)	
2015	539 (2.5)	15 334 (69.9)	6059 (27.6)	21932 (13.8)	
2016	576 (2.7)	15 108 (71.1)	5570 (26.2)	21254 (13.4)	
2017	601 (2.8)	15 055 (71.3)	5470 (25.9)	21126 (13.3)	
Age, y					
Median (IQR)	51 (54-66)	58 (51-64)	58 (50-64)	58 (50-64)	<.001
30-39	109 (1.2)	6333 (67.2)	2989 (31.7)	9431 (6.0)	<.001
40-49	425 (1.6)	18 104 (68.3)	7967 (30.1)	26 497 (16.7)	
50-59	1163 (2.2)	36 174 (69.7)	14 562 (28.1)	51 898 (32.8)	
≥60	2196 (3.1)	49 289 (69.8)	19 134 (27.1)	70 619 (44.6)	
Sex					
Men	2634 (2.7)	70 766 (71.1)	26 163 (26.3)	99 563 (62.8)	<.001
Women	1258 (2.1)	39 135 (66.5)	18 489 (31.4)	58 882 (37.2)	
Race/ethnicity					
White	2651 (2.2)	83 824 (69.9)	33 526 (28)	120 001 (75.7)	<.001
Black	742 (3.7)	12 966 (65.2)	6182 (31.1)	20 889 (13.2)	
Hispanic	386 (3.1)	8381 (68.3)	3505 (28.6)	12 271 (7.7)	
Other^d^	115 (2.2)	3730 (70.6)	1439 (27.2)	5283 (3.3)	
CCI score					
0	2910 (2.4)	84 334 (69.3)	34 478 (28.3)	121 721 (76.8)	.001
1	983 (2.7)	25 567 (69.6)	10 173 (27.7)	36 723 (23.2)	
Insurance status					
Private insurance	1437 (1.6)	64 085 (70.3)	25 695 (28.2)	91 218 (57.6)	<.001
No insurance	288 (4.7)	4487 (72.8)	1386 (22.5)	6161 (3.9)	
Medicaid	519 (4.1)	8723 (69.0)	3409 (26.9)	12 650 (8.0)	
Medicare	1556 (3.4)	30 697 (67.3)	13 368 (29.3)	45 620 (28.8)	
Other government insurance	94 (3.4)	1909 (68.3)	794 (28.4)	2796 (1.8)	
Facility type					
Community cancer program	371 (3.5)	7085 (67.6)	3023 (28.9)	10 479 (6.6)	<.001
Comprehensive community cancer center	1399 (2.5)	38 282 (68.0)	16 619 (29.5)	56 299 (35.5)	
Academic or research program	1586 (2.3)	49 656 (70.6)	19 081 (27.1)	70 323 (44.4)	
Integrated network cancer program	538 (2.5)	14 878 (69.7)	5928 (27.8)	21 344 (13.5)	
Facility annual patient volume					
Median (IQR)	52 (25-102)	64 (33-121)	60 (32-110)	63 (32-118)	<.001
Quartile (by year)					
First	222 (5.4)	2770 (67.9)	1088 (26.7)	4080 (2.6)	<.001
Second	538 (3.6)	10 163 (67.3)	4394 (29.1)	15 092 (9.5)	
Third	880 (2.6)	22 788 (67.9)	9890 (29.5)	33 568 (21.2)	
Fourth	2255 (2.1)	74 180 (70.2)	29 270 (27.7)	105 705 (66.7)	
Facility location					
Northeast	614 (1.9)	22 432 (69.9)	9037 (28.2)	32 083 (20.3)	<.001
South or Southeast	1711 (2.7)	42 781 (68.5)	17 963 (28.8)	62 455 (39.4)	
Midwest	893 (2.2)	28 419 (69.8)	11 400 (28.0)	40 711 (25.7)	
Mountain or West	675 (2.9)	16 269 (70.1)	6251 (27.0)	23 195 (14.6)	
Urban vs rural location					
Metropolitan	3208 (2.4)	91 229 (69.2)	37 375 (28.4)	131 812 (83.2)	.003
Urban	608 (2.6)	16 633 (70.3)	6437 (27.2)	23 678 (14.9)	
Rural	76 (2.6)	2039 (69.0)	839 (28.4)	2955 (1.9)	
Distance to facility					
Median (IQR), miles	11.4 (4.2-34.1)	13.9 (5.4-38.9)	13.1 (5.2-34.3)	13.6 (5.3-37.5)	<.001
Quartile					
First	1181 (2.9)	27 550 (68.3)	11 585 (28.7)	40 316 (25.4)	<.001
Second	972 (2.5)	27 221 (68.6)	11 500 (29.0)	39 692 (25.1)	
Third	859 (2.2)	27 391 (69.0)	11 462 (28.9)	39 712 (25.1)	
Fourth	881 (2.3)	27 740 (71.6)	10 105 (26.1)	38 725 (24.4)	
Median income by zip code, US $					
<40 227	989 (3.2)	21 405 (68.6)	8833 (28.3)	31 226 (19.7)	<.001
40 227-50 353	966 (2.7)	24 729 (69.6)	9814 (27.6)	35 509 (22.4)	
50 354-63 332	923 (2.5)	25 431 (69.1)	10 442 (28.4)	36 796 (23.2)	
≥63 333	1016 (1.9)	38 336 (69.8)	15 561 (28.3)	54 913 (34.7)	
% Without high school degree, by zip code					
≥17.6%	1147 (3.2)	24 762 (68.7)	10 145 (28.1)	36 054 (22.8)	<.001
10.9%-17.5%	1113 (2.6)	29 260 (69.1)	11 966 (28.3)	42 340 (26.7)	
6.3%-10.8%	979 (2.2)	30 785 (69.8)	12 333 (28.0)	44 097 (27.8)	
<6.3%	653 (1.8)	25 094 (69.8)	10 207 (28.4)	35 954 (22.7)	
Tumor size, cm					
0-2	0	2999 (10.1)	26 816 (90.0)	29 815 (18.8)	NA
>2-4	0	46 732 (72.4)	17 836 (27.6)	64 567 (40.8)	
>4-7	2969 (7.1)	38 830 (92.9)	0	41 799 (26.4)	
>7	924 (4.2)	21 320 (95.9)	0	22 264 (14.1)	
AJCC clinical T stage					
cT1	2877 (2.3)	81 510 (65.3)	40 427 (32.4)	124 814 (78.8)	NA
cT2	1016 (3.0)	28 391 (84.4)	4224 (12.6)	33 631 (21.2)	
Treatment modality					
Observation or surveillance	3396 (29.0)	8333 (71.0)	0	11 729 (7.4)	NA
Ablation	496 (5.5)	5209 (57.4)	3363 (37.1)	9067 (5.7)	
Nephrectomy					
Partial	0	50 555 (72.6)	19 038 (27.4)	69 593 (43.9)	
Radical	0	45 804 (67.3)	22 251 (32.7)	68 055 (43.0)	

Abbreviations: AJCC, American Joint Committee on Cancer; CCI, Charlson-Deyo Comorbidity Index; IQR, interquartile range; NA, not applicable.

^a^ Percentages are row percentages for the undertreatment, guideline-based treatment, and overtreatment columns and column percentages for the total column.

^b^ Reported *P* values are per Kruskal-Wallis 1-way analysis of variance for continuous variables and Pearson χ^2^ test for categorical variables. *P* values are not reported for clinical factors or treatment modality given that these factors constitute the definition of undertreatment and overtreatment.

^c^ Percentages for years under treatment categories are out of year totals. Percentages for years under total column are out of overall total.

^d^ The other group includes Asian or Pacific Islander and American Indian or Alaskan native individuals, as well as those without a reported race/ethnicity.

### Sex

Multinomial logistic regression analysis of demographic and institutional factors associated with receipt of non-guideline–based treatment is shown in Table 2. In the overall study population, women were treated more aggressively than men, with statistically significantly lower adjusted odds of undertreatment (odds ratio [OR], 0.82; 95% CI, 0.77-0.88; *P* < .001) and statistically significantly higher adjusted odds of overtreatment (OR, 1.27; 95% CI, 1.24-1.30; *P* < .001), controlling for other demographic factors (Table 2). In subgroup analysis, the association between female sex and overtreatment was preserved for small (ie, <2 cm) cT1a kidney masses (OR, 1.15; 95% CI, 1.06-1.24; *P* = .001) and large (ie, 2-4 cm) cT1a kidney masses (OR, 1.13; 95% CI, 1.05-1.21; *P* < .001) (Figure). Regarding undertreatment of aggressive tumors, women had statistically significantly lower adjusted odds of undertreatment for cT1b tumors (OR, 0.79; 95% CI, 0.73-0.86; *P* < .001). However, no statistically significant association was observed for cT2 tumors. Together, these observations show an association of female sex with more aggressive management of lower-stage kidney masses compared with male sex.

**Table 2.** Multinomial Logistic Regression of Demographic Factors

Characteristic^a^	Undertreatment (multivariable)		Overtreatment (multivariable)	
	OR (95% CI)	*P* value	OR (95% CI)	*P* value
Age, per year	1.03 (1.02-1.03)	<.001	0.99 (0.99-0.99)	<.001
Year of diagnosis, each year	1.03 (1.01-1.04)	<.001	0.96 (0.96-0.97)	<.001
Sex				
Men	1 [Reference]	NA	1 [Reference]	<.001
Women	0.82 (0.77-0.88)	<.001	1.27 (1.24-1.30)	
Race/ethnicity				
White	1 [Reference]	NA	1 [Reference]	NA
Black	1.42 (1.29-1.55)	<.001	1.09 (1.05-1.13)	<.001
Hispanic	1.20 (1.06-1.36)	.004	1.06 (1.01-1.11)	.01
CCI score				
0	1 [Reference]	NA	1 [Reference]	NA
1	1.00 (0.93-1.08)	.98	0.97 (0.94-1.00)	.02
Insurance status				
Private insurance	1 [Reference]	NA	1 [Reference]	NA
No insurance	2.63 (2.29-3.02)	<.001	0.72 (0.67-0.77)	<.001
Medicaid	2.44 (2.19-2.73)	<.001	0.95 (0.91-0.99)	.03
Medicare	1.67 (1.53-1.82)	<.001	1.21 (1.17-1.24)	<.001
Facility type				
Community cancer program	1 [Reference]	NA	1 [Reference]	NA
Comprehensive community cancer center	1.08 (0.94-1.25)	.28	1.03 (0.97-1.09)	.31
Academic or research program	1.14 (0.98-1.34)	.09	0.94 (0.88-1.00)	.05
Integrated network cancer program	1.09 (0.93-1.27)	.30	0.95 (089-1.01)	.11
Facility annual patient volume, quartile				
First	1 [Reference]	NA	1 [Reference]	NA
Second	0.64 (0.53-0.76)	<.001	1.10 (1.01-1.19)	.03
Third	0.47 (0.39-0.56)	<.001	1.11 (1.02-1.21)	.02
Fourth	0.37 (0.31-0.44)	<.001	1.06 (0.97-1.15)	.20
Facility location				
Northeast	1 [Reference]	NA	1 [Reference]	NA
South or Southeast	1.33 (1.20-1.48)	<.001	1.03 (1.00-1.07)	.08
Midwest	1.13 (1.01-1.26)	.04	1.00 (0.96-1.03)	.84
Mountain or West	1.45 (1.29-1.64)	<.001	0.94 (0.90-0.98)	.003
Urban vs rural location				
Metropolitan	1 [Reference]	NA	1 [Reference]	NA
Urban	1.01 (0.90-1.12)	.90	1.00 (0.96-1.04)	.88
Rural	1.00 (0.78-1.30)	.98	1.07 (0.98-1.17)	.13
Distance to facility, quartile				
First	1 [Reference]	NA	1 [Reference]	NA
Second	0.94 (0.86-1.03)	.18	1.01 (0.97-1.04)	.74
Third	0.86 (0.78-0.95)	.003	1.01 (0.97-1.04)	.72
Fourth	0.84 (0.75-0.95)	.004	0.89 (0.85-0.93)	<.001
Median income by zip code, US $				
<40 227	1 [Reference]	NA	1 [Reference]	NA
40 227-50 353	0.99 (0.88-1.10)	.80	0.98 (0.95-1.02)	.40
50 354-63 332	0.99 (0.88-1.12)	.93	1.02 (0.98-1.06)	.36
≥63 333	0.82 (0.72-0.95)	.006	1.02 (0.97-1.07)	.40
% Without high school degree, by zip code				
≥17.6%	1 [Reference]	NA	1 [Reference]	NA
10.9%-17.5%	0.94 (0.85-1.03)	.19	1.00 (0.97-1.04)	.82
6.3%-10.8%	0.89 (0.80-1.01)	.06	0.99 (0.95-1.03)	.54
<6.3%	0.84 (0.72-0.97)	.02	1.01 (0.96-1.06)	.64

Abbreviations: CCI, Charlson-Deyo Comorbidity Index; NA, not applicable; OR, odds ratio.

^a^ The regression model included all covariates shown.

**Figure.**
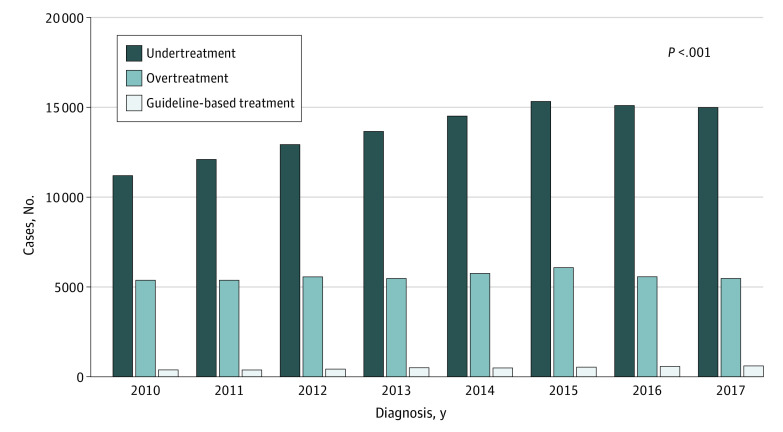
Multivariable Analysis of Factors Associated With Non-Guideline–Based Treatment by Tumor Category The following covariates were included in the regression models but are omitted here for brevity: age, year of diagnosis, Charlson-Deyo Comorbidity Index score, facility type, facility location, facility annual patient volume, urban vs rural location, distance traveled to facility, income, and educational attainment. Odds ratios (ORs) from the primary multinomial regression analysis of the overall population are included for comparison.

### Race/Ethnicity

Black race was associated with statistically significantly higher adjusted odds of undertreatment (OR, 1.42; 95% CI, 1.29-1.55; *P* < .001) and overtreatment (OR, 1.09; 95% CI, 1.05-1.13; *P* < .001) compared with White race, controlling for other demographic factors (Table 2). In subgroup analysis, the association of Black race with undertreatment was statistically significant for cT1b and cT2 kidney masses; among individuals with smaller masses, adjusted odds of overtreatment were statistically significantly lower for patients with small cT1a masses (OR, 0.72; 95% CI, 0.64-0.80; *P* < .001) and adjusted odds of overtreatment were statistically significantly higher for individuals with larger masses (OR, 1.09; 95% CI, 1.03-1.15; *P* = .003) (Figure). Hispanic ethnicity was associated with undertreatment (OR, 1.20; 95% CI, 1.06-1.36; *P* = .004) and overtreatment (OR, 1.06; 95% CI, 1.01-1.11; *P* = .01) in the primary analysis (Table 2), but among subgroups, this association was significant in only some tumor classifications (Figure).

### Insurance Status

Compared with having private insurance, there was a significant association between uninsured status and receiving less aggressive treatment. Uninsured status was associated with statistically significantly higher adjusted odds of undertreatment (OR, 2.63; 95% CI, 2.29-3.01; *P* < .001) and lower adjusted odds of overtreatment (OR, 0.72; 95% CI, 0.67-0.77; *P* < .001) (Table 2). In subgroup analysis, these associations were significant for overtreatment of small cT1a masses (OR, 0.40; 95% CI, 0.33-0.49; *P* < .001) (Figure) and undertreatment of cT1b masses (OR, 2.49; 95% CI, 2.10-2.94; *P* < .001) and cT2 masses (OR, 2.77; 95% CI, 2.11-3.62; *P* < .001) but not for overtreatment of large cT1a masses (OR, 1.01; 95% CI, 0.91-1.11; *P* = .92) (Figure). A similar pattern was noted for patients with Medicaid (overtreatment of small cT1a masses: OR, 0.58; 95% CI, 0.50-0.67; *P* < .001; undertreatment of cT1b masses: OR, 2.44; 95% CI, 2.14-2.78; *P* < .001; undertreatment of cT2 masses: OR, 2.89; 95% CI, 2.31-3.61; *P* < .001; overtreatment of large cT1a masses: OR, 1.04; 95% CI, 0.97-1.12; *P* = .24). Having Medicare was associated with statistically significantly higher adjusted odds of undertreatment (OR, 1.67; 95% CI, 1.53-1.82; *P* < .001) and overtreatment (OR, 1.21; 95% CI, 1.17-1.24; *P* < .001) (Table 2). The higher adjusted odds of overtreatment appeared to be associated with higher adjusted odds of overtreatment of large cT1a masses (OR, 1.20; 95% CI, 1.14-1.26; *P* < .001).

### Sensitivity Analysis

As a sensitivity analysis, the primary multinomial logistic regression was repeated on a separate, nonimputed data set in which patients with incomplete data on tumor stage and size or treatment were excluded (eTable 4 in the Supplement). Subgroup analyses were repeated with the inclusion of tumor size as a continuous covariate (eTable 5 in the Supplement). Similar outcomes were observed as in the primary analysis.

## Discussion

This cohort study found that female, Black, and Hispanic patients had higher adjusted odds of non-guideline–based treatment for localized kidney cancer and, notably, that Black and Hispanic patients had higher adjusted odds of both undertreatment and overtreatment. The diagnosis and management of kidney cancer has undergone dramatic shifts in the past several decades. Widespread use of cross-sectional imaging has led to an increase in incidental diagnosis of early stage kidney cancers, and patient outcomes have improved over this period in association with stage migration as well as new systemic treatments for advanced disease.^1,13,14^ However, it is increasingly recognized that overtreatment of small, indolent kidney masses is associated with significant morbidity in itself and may be harmful to patients.^2,3,15^ Robotic-assisted surgical technology has been widely adopted and is associated with decreased treatment morbidity and increased use of nephron-sparing approaches; however, the availability of robotic technology may itself be associated with overtreatment of small kidney masses.^16,17,18^

Despite the increasing attention devoted to overtreatment of indolent malignant tumors in urologic oncology, many patients with aggressive disease are undertreated, particularly members of racial/ethnic minority groups. Numerous studies have found adverse outcomes among patients with kidney cancer who are Black or members of other racial/ethnic minority groups compared with White patients.^7,9,19,20,21^ Two 2020 studies^22,23^ found that Black patients with kidney cancer were less likely to be referred to high-volume surgical centers than other patients and that hospitals serving patients from racial/ethnic minority groups were less likely to offer surgical treatment. Similar phenomena have been described in other genitourinary malignant tumors. For example, in 1 series, young and healthy Black and Hispanic patients with high-risk prostate cancer were less likely than White patients to be treated with curative intent.^24^ In a series of older patients with prostate cancer, Black patients were more likely than White patients to have delayed treatment and to incur higher treatment costs and had higher odds of readmission.^25^ In a state-level series, Black patients with bladder cancer were more likely to be treated at low-volume centers, although this disparity did decline over time with increasing centralization of care.^26^ Disparities in cancer treatment have also been associated with uninsured or underinsured status; for example, patients with kidney cancer who were uninsured or had Medicaid were more likely to present with advanced disease, less likely to receive definitive treatment, and more likely to die of disease than those with private insurance.^8^ Additionally, although disparities in cancer outcomes between the sexes are often attributed to underlying biological factors, there may be systematic inequalities between the sexes in access to care or in treatment decision-making. For example, inappropriate underevaluation of female patients with hematuria has been postulated as a cause of poorer outcomes in female patients with bladder cancer.^27^

In this study, we performed a retrospective analysis of the NCDB to assess the interaction of demographic factors with decisions to treat patients who had localized kidney tumors more or less aggressively. To minimize confounding effects of age and comorbidity, we confined our analysis to a young and healthy subset of patients. We categorized patients as having received guideline-based treatment, overtreatment, or undertreatment based on NCCN guidelines.

Female sex was associated with significantly lower odds of undertreatment and higher odds of overtreatment. This observation is interesting in light of prior literature suggesting underreferral and undertreatment of women presenting with hematuria, as well as undertreatment of female patients with other malignant tumors, including head and neck cancer.^27,28^ One might question whether there are underlying clinician-driven or patient-driven reasons for this disparity. For example, there may be a tendency of clinicians to perceive female patients as having greater potential longevity and therefore warranting more aggressive cancer treatment. Alternately or concurrently, there may be a systematic preference among female patients for more aggressive treatment.

With regard to race/ethnicity, Black patients had higher odds than White patients of receiving non-guideline–based treatment, consistent with previously reported racial/ethnic discrepancies in surgical management of kidney cancer.^22,23^ Interestingly, Black race was associated with higher odds of both undertreatment and overtreatment, and this association was significant across all subgroups, with the exception of the group with the smallest cT1a tumors, in which Black patients had lower odds of overtreatment. Systematic undertreatment of Black patients has been described in other malignant tumors, including prostate cancer.^24^ However, our observation that Black patients also had higher odds of overtreatment for kidney cancer is novel and highlights the complexity of the individual patient-level decision-making processes that underlie systemic inequities. For example, overtreatment may result from limited physical resources (eg, lack of access to a surgical robot, rendering partial nephrectomy less appealing), limited human resources (eg, lack of a surgeon who is familiar with partial nephrectomy), or a higher level of real or perceived patient comorbidity resulting in a preference for radical nephrectomy over partial nephrectomy. The higher overtreatment odds appeared to be associated primarily with the large (ie, 2-4 cm) cT1a subgroup, suggesting increased use of radical nephrectomy in small but clinically significant masses that might otherwise be appropriate for partial nephrectomy.

We noted a complex association between insurance status and receipt of non-guideline–based treatment. Uninsured status was associated with lower odds of overtreatment of small cT1a tumors and higher odds of undertreatment compared with private insurance, in a general trend toward less aggressive treatment. As mentioned above, this phenomenon may be associated, at least in part, with decision-making in resource-limited settings; for example, a patient with a large cT1a tumor in a hospital that lacks a surgical robot may be more likely to undergo a radical rather than a partial nephrectomy. One notable observation was that there were higher odds of overtreatment associated with Medicare insurance, associated in part with higher rates of overtreatment for large cT1a masses. It should be noted that patients with Medicare in this cohort represented a select group. Our inclusion criteria enriched for patients with Medicare under age 65 years, and eligibility for Medicare in this population requires a qualifying disability (eg, end-stage kidney disease, amyotrophic lateral sclerosis, or ≥24 months of Social Security disability payments). Our eligibility criteria would be expected to exclude patients with major comorbidities; however, it is possible that the overtreatment outcome seen here was partially associated with decisions to perform radical nephrectomy in patients with end-stage kidney disease who had moderate size tumors in nonfunctional kidneys.

### Limitations

Several limitations of our study should be noted. It should be acknowledged that clinical guidelines may not represent the ideal treatment for all patients, and the decision to treat a patient more or less aggressively than recommended by guidelines may be entirely appropriate. It should also be acknowledged that we retrospectively applied a contemporary guideline to patients treated in the past. However, the NCCN guideline used here broadly mirrors the recommendations of the 2009 AUA guideline statement, which was in effect for the entirety of the study period.^11^ While there may be concerns about the application of our overtreatment and undertreatment classification to individual patients, we believe the classification represents a reasonable metric for determining the aggressiveness of cancer treatment decisions across large groups of patients.

With regard to other limitations, the NCDB relies on registry data submitted by CoC-accredited cancer centers, and its underlying accuracy cannot be ascertained. Systematic inaccuracies may be present. In particular, race/ethnicity as reported in large databases does not correlate perfectly with patients’ self-reports, and such discrepancies could be a source of systematic error in our findings.^29^ The NCDB is not a population-based database, and while it captures more than 70% of new cancer diagnoses in the United States, it does represent a nonrandom subset of the overall population. Our study population may not represent a comprehensive sample of patients presenting for evaluation of kidney masses, given that individuals with smaller masses who undergo surveillance may not be captured by the database owing to nonreferral. We made efforts to minimize the effects of comorbidity and clinical factors by excluding patients who were older, had comorbidities, or had advanced-stage disease from our analysis; however, unaccounted confounding factors may remain.

Additionally, although we found that several demographic and institutional factors were associated with receipt of non-guideline–based treatment for kidney cancer, we are unable to determine the reasons underlying these disparities or to assess the associations of these disparities with clinical outcomes. Our study carries the limitations of a retrospective study, including the inability to attribute causality to the associations we found.

## Conclusions

To our knowledge, this study represents the first attempt to assess the associations between demographic factors and receipt of non-guideline–based treatment among patients with kidney cancer. We found that female patients with kidney cancer had higher odds of receiving more aggressive treatment than men, which was associated with increased rates of overtreatment for small kidney masses and potentially increased risk for unjustified complications. Black race and Hispanic ethnicity were associated with higher odds of undertreatment and overtreatment, highlighting the bidirectional nature of inequities in treatment. Patients without insurance had markedly lower odds of overtreatment for very small kidney masses, potentially representing a rare example of a salutary association of reduced access to care. Clinicians should bear these disparities in mind when counseling individual patients, and health policy makers should take the existence of these disparities into account.

## Notes

Supplement. **eAppendix.** Supplemental Methods eReferences **eTable 1.** Proportion of Missing Values **eTable 2.** Definitions of Tumor Size Classifications and Corresponding Guideline-Based Treatments **eTable 3.** Variance Inflation Factors for the Primary Multinomial Regression Analysis and for Each Subgroup Analysis **eTable 4.** Sensitivity Analysis for Primary Outcome **eTable 5.** Subgroup Sensitivity Analysis With Tumor Size as a Continuous Covariate **eFigure.** Inclusion and Exclusion Criteria
