# Supplementary material for: Demographic Factors Associated With Non-Guideline–Based Treatment of Kidney Cancer in the United States
Source: JAMA Netw Open. 2021 Jun 9;4(6):e2112813. doi: 10.1001/jamanetworkopen.2021.12813 (PMC8190623; doi:10.1001/jamanetworkopen.2021.12813)
Supplement: Supplement. — eAppendix. Supplemental Methods eReferences eTable 1. Proportion of Missing Values eTable 2. Definitions of Tumor Size Classifications and Corresponding Guideline-Based Treatments eTable 3. Variance Inflation Factors for the Primary Multinomial Regression Analysis and for Each Subgroup Analysis eTable 4. Sensitivity Analysis for Primary Outcome eTable 5. Subgroup Sensitivity Analysis With Tumor Size as a Continuous Covariate eFigure. Inclusion and Exclusion Criteria [file jamanetwopen-e2112813-s001.pdf]

# Supplemental Online Content

Howard JM, Nandy K, Woldu SL, Margulis V. Demographic factors associated with non-guideline–based treatment of kidney cancer in the United States. *JAMA Netw Open*. 2021;4(6):e2112813. doi:10.1001/jamanetworkopen.2021.12813

**eAppendix.** Supplemental Methods
**eReferences**
**eTable 1.** Proportion of Missing Values
**eTable 2.** Definitions of Tumor Size Classifications and Corresponding Guideline-Based Treatments
**eTable 3.** Variance Inflation Factors for the Primary Multinomial Regression Analysis and for Each Subgroup Analysis
**eTable 4.** Sensitivity Analysis for Primary Outcome
**eTable 5.** Subgroup Sensitivity Analysis With Tumor Size as a Continuous Covariate
**eFigure.** Inclusion and Exclusion Criteria

This supplemental material has been provided by the authors to give readers additional information about their work.

© 2021 Howard JM et al. *JAMA Network Open.*

**eAppendix.** Supplemental Methods

*Covariates*

Covariates included in the analysis were patient age, sex, race/ethnicity, Charlson-Deyo comorbidity index, insurance status (private insurance, uninsured, Medicaid, Medicare, other government insurance), facility type (community cancer program, comprehensive community cancer program, academic/research program, facility location (Northeast, South/Southeast, Midwest, Mountain/West), urban/rural facility setting (metro, urban, rural), great-circle distance from patient's residence to facility, annual institutional case volume, median income and educational attainment of patient's home ZIP code, and year of diagnosis. Patient sex and race/ethnicity were abstracted from medical records by the participating institutions and were therefore dependent on documentation practices of the individual treating institutions, which could not be determined with certainty. Race/ethnicity was classified as White, Black, Hispanic, or other. Patients of any race reported as having Hispanic ethnicity were classified as Hispanic. Treatment modality was categorized as observation/surveillance (including non-curative treatments), ablation, partial nephrectomy, and radical nephrectomy. Patients with a delay from diagnosis to treatment of ≥ 6 months were deemed to have undergone a trial of observation. Institutional case volume was calculated as the number of kidney cancer cases reported by a given institution in a given year. Institutional case volumes were stratified into quartiles by year. Missing tumor sizes were inferred from the clinical T stage, where possible. Conflicts between reported tumor size and clinical T1-T2 stage were resolved in favor of the reported tumor size. Tumor histology was stratified into clear cell, papillary, chromophobe, sarcomatoid, collecting duct, medullary carcinoma, and mixed/unspecified/other for the multiple imputation. Due to small case numbers the sarcomatoid, collecting duct, and medullary histologies were combined with the mixed/unspecified/other group when reporting.

© 2021 Howard JM et al. *JAMA Network Open.*

*Multiple Imputation of Missing Values*

As discussed in the main text, the data set was first filtered by the inclusion criteria of year of diagnosis, age, and Charlson-Deyo score, none of which had missing values, to yield a group of 214,763 patients. Cases with metastatic disease (*n* = 27,717) were excluded using variables reporting the presence of metastases at diagnosis and the reported clinical and pathologic M stages. A small minority of cases (*n* = 1,559, 0.7%) with no data on metastatic status were also excluded. Finally, a small group of patients with documented cT0 stage (*n* = 196) were excluded, yielding a population of 185,291 patients with non-metastatic kidney tumors. Tumor histology was not used as an inclusion criterion as this would not necessarily be known at the time of treatment decision making.

Analysis of missing values indicated substantial proportions of missing clinical and demographic data (eTable 1). The pattern of missingness was not monotone. Missing values were imputed 20 times in 10 iterations using fully conditional specification. The imputation model included the following as both independent and imputed variables: facility type, facility location, urban/rural status, race/ethnicity, insurance status, income by ZIP code, educational attainment by ZIP code, great-circle distance to facility, tumor size (categorical), and clinical T and N stages. Independent variables which were not imputed included age, sex, Charlson-Deyo score, year of diagnosis, annual institutional patient volume by quartile, tumor histology, pathologic T and N stages, clinical and pathologic summary stages, and NCDB analytic stage group. Treatment modality was missing in a minority (1.0%) of cases. As an outcome variable, it was imputed where missing but was not included as an independent factor in the imputation. Statistical analyses were performed separately on each imputed data set and the results were then pooled, using Rubin's rules for parametric tests and taking the median of calculated values for nonparametric tests, for which Rubin's rules are not applicable. Significance across multiple imputed data sets was determined using a similar technique[1].

© 2021 Howard JM et al. *JAMA Network Open.*

*Validations of Multivariable Analyses*

For all multivariable analyses, multicollinearity of the included covariates was assessed by calculation of variance inflation factors (VIFs). With one minor exception, VIFs were ≤ 2.0, suggesting a minimal effect of multicollinearity on the outcome (eTable 3). For the primary multinomial analysis of undertreatment versus overtreatment versus guideline-based treatment, a sensitivity analysis was performed on an alternate, non-imputed data set in which cases with missing information were excluded. This yielded similar results to the primary analysis (eTable 4). Tumor size was not included as a covariate in the primary multinomial regression analysis, as it was part of the definition of guideline-based treatment. However, it was recognized that tumor size could vary within tumor subgroups and that this could confound the results. We therefore repeated the subgroup (binomial regression) analyses including tumor size as a continuous covariate. This did not significantly change the result (eTable 5).

© 2021 Howard JM et al. *JAMA Network Open.*

## eReferences

1. Eekhout I, Van De Wiel MA and Heymans MW: Methods for significance testing of categorical covariates in logistic regression models after multiple imputation: power and applicability analysis. *BMC Med Res Methodol* 2017;17:1–12.

© 2021 Howard JM et al. *JAMA Network Open.*

**eTable 1.** Proportion of Missing Values

| **Characteristic** | **Missing Values** ***n*** **(%)** |
|---|---|
| total patients | 185,291 |
| age | 0 |
| sex | 0 |
| race/ethnicity | 1,557 (0.8%) |
| Charlson-Deyo comorbidity index | 0 |
| insurance status | 3,579 (1.9%) |
| facility type | 10,621 (5.7%) |
| facility annual patient volume, median (IQR) | 0 |
| facility location | 10,621 (5.7%) |
| urban/rural location | 6,252 (3.4%) |
| distance to facility, median (IQR), miles | 20,594 (11.1%) |
| median income by ZIP code, US$ | 22,611 (12.2%) |
| % without high school degree, by ZIP code | 22,326 (12.0%) |
| tumor size | 1,586 (0.9%) |
| AJCC clinical T stage | 31,404 (16.9%) |
| AJCC clinical N stage | 20,878 (11.3%) |
| treatment modality | 1,809 (1.0%) |

Among *n* = 185,291 patients with non-metastatic kidney cancer prior to multiple imputation.

© 2021 Howard JM et al. *JAMA Network Open.*

**eTable 2.** Definitions of Tumor Size Classifications and Corresponding Guideline-Based Treatments

| Tumor Classification | Undertreatment | Guideline-Based Treatment | Overtreatment |
|---|---|---|---|
| cT1a (< 2 cm) | | surveillance | ablation<br>partial nephrectomy<br>radical nephrectomy |
| cT1a (2 - 4 cm) | | surveillance<br>ablation<br>partial nephrectomy | radical nephrectomy |
| cT1b (4 - 7 cm) | surveillance<br>ablation | partial nephrectomy<br>radical nephrectomy | |
| cT2 (> 7 cm) | surveillance<br>ablation | partial nephrectomy<br>radical nephrectomy | |

© 2021 Howard JM et al. *JAMA Network Open.*

**eTable 3.** Variance Inflation Factors for the Primary Multinomial Regression Analysis and for Each Subgroup Analysis

| Covariate | Primary Analysis | Subgroup Analysis | | | |
|---|---|---|---|---|---|
| | | Small cT1a | Large cT1a | cT1b | cT2 |
| age | 1.21 | 1.19 | 1.20 | 1.23 | 1.23 |
| year of diagnosis | 1.00 | 1.01 | 1.01 | 1.00 | 1.00 |
| sex | 1.00 | 1.00 | 1.01 | 1.01 | 1.00 |
| race/ethnicity | 1.10 | 1.09 | 1.09 | 1.10 | 1.10 |
| Charlson-Deyo comorbidity index | 1.02 | 1.02 | 1.02 | 1.02 | 1.02 |
| insurance status | 1.21 | 1.20 | 1.21 | 1.23 | 1.22 |
| facility type | 1.20 | 1.20 | 1.19 | 1.21 | 1.20 |
| facility location | 1.02 | 1.02 | 1.02 | 1.02 | 1.02 |
| institutional case volume | 1.24 | 1.23 | 1.23 | 1.25 | 1.24 |
| urban/rural status | 1.34 | 1.32 | 1.33 | 1.35 | 1.36 |
| distance traveled to facility | 1.27 | 1.25 | 1.26 | 1.28 | 1.30 |
| median income | 1.98 | 2.01 | 1.97 | 1.96 | 1.97 |
| median educational attainment | 1.91 | 1.94 | 1.91 | 1.90 | 1.89 |

Values shown are the median of variance inflation factors (VIFs) calculated separately for 20 imputed data sets.

© 2021 Howard JM et al. *JAMA Network Open.*

**eTable 4.** Sensitivity Analysis for Primary Outcome

| ***Analysis*** **Characteristic** | **Undertreatment (Multivariable)** | | | **Overtreatment (Multivariable)** | | |
|---|---|---|---|---|---|---|
| | **OR** | **95% CI** | ***p*** | **OR** | **95% CI** | ***p*** |
| sex | | | | | | |
| male | reference | | | reference | | |
| female | **0.81** | **0.75 - 0.88** | **< 0.001** | **1.28** | **1.25 - 1.32** | **< 0.001** |
| race/ethnicity | | | | | | |
| White | reference | | | reference | | |
| Black | **1.40** | **1.26 - 1.55** | **< 0.001** | **1.06** | **1.02 - 1.11** | **0.006** |
| Hispanic | **1.21** | **1.06 - 1.39** | **0.005** | 1.05 | 1.00 - 1.11 | 0.067 |
| insurance status | | | | | | |
| private insurance | reference | | | reference | | |
| uninsured | **2.58** | **2.20 - 3.02** | **< 0.001** | **0.73** | **0.67 - 0.79** | **< 0.001** |
| Medicaid | **2.43** | **2.15 - 2.75** | **< 0.001** | **0.93** | **0.88 - 0.98** | **0.010** |
| Medicare | **1.67** | **1.51 - 1.83** | **< 0.001** | **1.18** | **1.14 - 1.22** | **< 0.001** |

Multinomial logistic regression analysis using the original, non-imputed data set with cases missing tumor size or treatment information excluded (*n* = 138,016 cases with complete information). The following covariates were included in the model but are not shown here for brevity: age, year of diagnosis, Charlson-Deyo comorbidity index, facility type, facility location, urban/rural location, facility annual patient volume, distance traveled to facility, income and educational attainment by patient's home ZIP code. Comparisons meeting the threshold for significance ($p \leq 0.05$) are highlighted in **bold**.

© 2021 Howard JM et al. *JAMA Network Open.*

**eTable 5.** Subgroup Sensitivity Analysis With Tumor Size as a Continuous Covariate

| ***Tumor Category*** **Characteristic** | **Overtreatment (Multivariable)** | | |
|---|---|---|---|
| | **OR** | **95% CI** | ***p*** |
| *small cT1a (< 2 cm)* | | | |
| sex | | | |
| male | reference | | |
| female | **1.15** | **1.06 - 1.25** | **0.001** |
| race/ethnicity | | | |
| White | reference | | |
| Black | **0.72** | **0.64 - 0.80** | **< 0.001** |
| Hispanic | 0.91 | 0.78 - 1.06 | 0.22 |
| insurance status | | | |
| private insurance | reference | | |
| uninsured | **0.40** | **0.33 - 0.49** | **< 0.001** |
| Medicaid | **0.58** | **0.50 - 0.67** | **< 0.001** |
| Medicare | **0.79** | **0.72 - 0.87** | **< 0.001** |
| tumor size (per 1 cm) | 1.10 | 1.00 - 1.21 | 0.055 |
| *large cT1a (2 - 4 cm)* | | | |
| sex | | | |
| male | reference | | |
| female | **1.16** | **1.11 - 1.20** | **< 0.001** |
| race/ethnicity | | | |
| White | reference | | |
| Black | **1.11** | **1.05 - 1.17** | **< 0.001** |
| Hispanic | **1.13** | **1.05 - 1.21** | **0.002** |
| insurance status | | | |
| private insurance | reference | | |
| uninsured | 0.98 | 0.87 - 1.09 | 0.72 |
| Medicaid | 1.03 | 0.96 - 1.11 | 0.38 |
| Medicare | **1.21** | **1.15 - 1.27** | **< 0.001** |
| tumor size (per 1 cm) | **2.23** | **2.16 - 2.31** | **< 0.001** |

| ***Tumor Category*** **Characteristic** | **Undertreatment (Multivariable)** | | |
|---|---|---|---|
| | **OR** | **95% CI** | ***p*** |
| *cT1b (2 - 7 cm)* | | | |
| sex | | | |
| male | reference | | |
| female | **0.78** | **0.72 - 0.85** | **< 0.001** |
| race/ethnicity | | | |
| White | reference | | |
| Black | **1.47** | **1.31 - 1.64** | **< 0.001** |
| Hispanic | **1.21** | **1.05 - 1.41** | **0.009** |
| insurance status | | | |
| private insurance | reference | | |
| uninsured | **2.53** | **2.14 - 3.00** | **< 0.001** |
| Medicaid | **2.44** | **2.14 - 2.78** | **< 0.001** |
| Medicare | **1.72** | **1.55 - 1.90** | **< 0.001** |
| tumor size (per 1 cm) | **0.74** | **0.71 - 0.78** | **< 0.001** |
| *cT2 (> 7 cm)* | | | |
| sex | | | |
| male | reference | | |
| female | 0.99 | 0.85 - 1.15 | 0.87 |
| race/ethnicity | | | |
| White | reference | | |

© 2021 Howard JM et al. *JAMA Network Open.*

| *Tumor Category* **Characteristic** | **Undertreatment (Multivariable)** | | |
|---|---|---|---|
| | **OR** | **95% CI** | ***p*** |
| Black | **1.65** | **1.37 - 1.99** | **< 0.001** |
| Hispanic | 1.21 | 0.94 - 1.56 | 0.14 |
| insurance status | | | |
| private insurance | reference | | |
| uninsured | **2.78** | **2.12 - 3.64** | **< 0.001** |
| Medicaid | **2.89** | **2.31 - 3.61** | **< 0.001** |
| Medicare | **1.96** | **1.63 - 2.36** | **< 0.001** |
| tumor size (per 1 cm) | **0.98** | **0.96 - 1.00** | **0.017** |

The following covariates were included in the model but are omitted here for brevity: age, year of diagnosis, Charlson-Deyo comorbidity index, facility type, facility location, facility annual patient volume, urban/rural location, distance traveled to facility, income, and educational attainment. Comparisons meeting the threshold for significance ($p \leq 0.05$) are highlighted in **bold**.

© 2021 Howard JM et al. *JAMA Network Open.*

**eFigure.** Inclusion and Exclusion Criteria

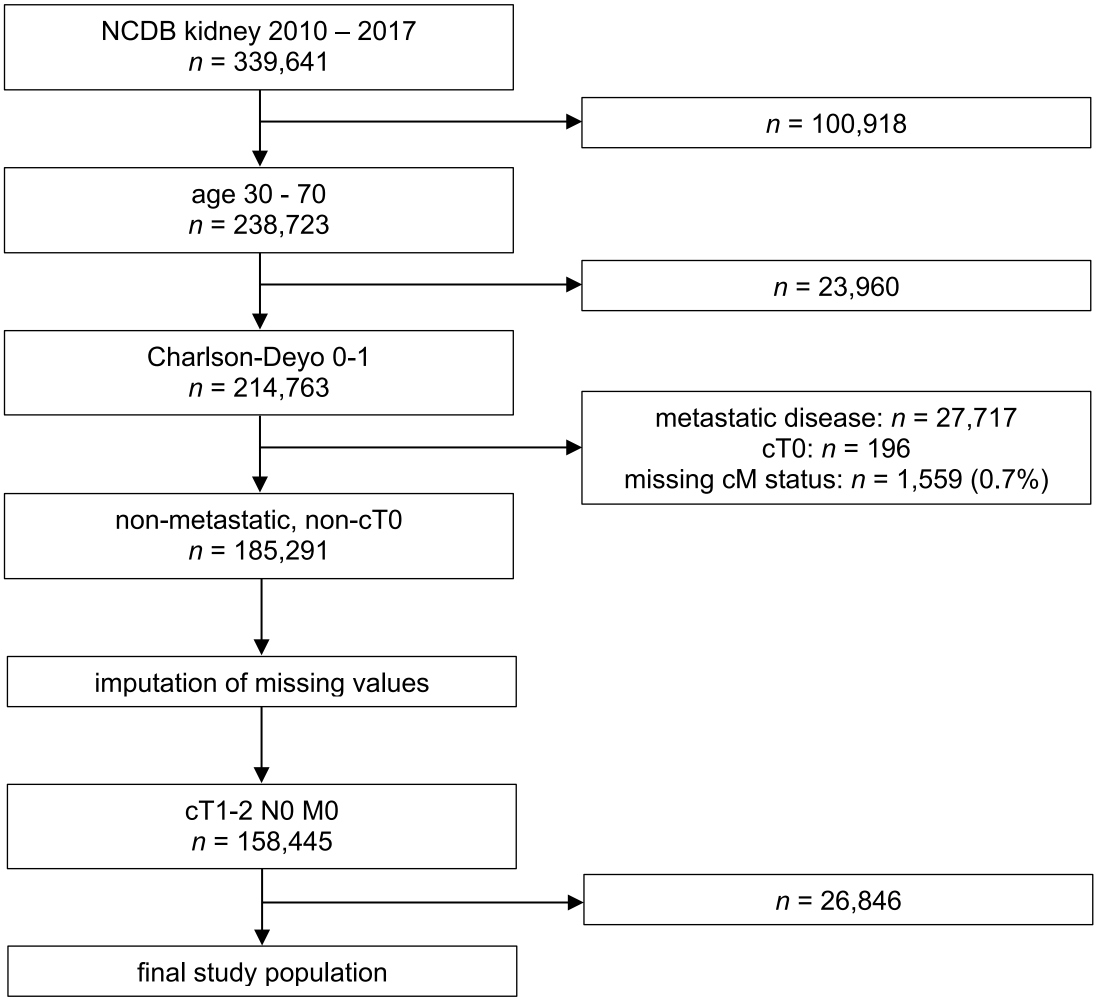


The final study population consisted of 158,445 patients aged 30 - 70 with cT1-2 N0 M0 kidney cancer.
Abbreviations; NCDB, National Cancer Database.

© 2021 Howard JM et al. *JAMA Network Open.*
